# Off hour admission to an intensivist-led ICU is not associated with increased mortality

**DOI:** 10.1186/cc7904

**Published:** 2009-06-05

**Authors:** Iwan A Meynaar, Johan I van der Spoel, Johannes H Rommes, Margot van Spreuwel-Verheijen, Rob J Bosman, Peter E Spronk

**Affiliations:** 1 ICU, Reinier de Graaf Groep, Reinier de Graafweg 3-11, 2625 AD, Delft, Netherlands; 2 ICU, Onze Lieve Vrouwe Gasthuis, Oosterpark 9, 1091 AC, Amsterdam, Netherlands; 3 ICU, Gelre Hospitals, Albert Schweitzerlaan 31 7334 DZ, Apeldoorn, Netherlands; 4 ICU, Academic Medical Centre, Meibergdreef 9, 1105 AZ, Amsterdam, Netherlands

## Abstract

**Introduction:**

Caring for the critically ill is a 24-hour-a-day responsibility, but not all resources and staff are available during off hours. We evaluated whether intensive care unit (ICU) admission during off hours affects hospital mortality.

**Methods:**

This retrospective multicentre cohort study was carried out in three non-academic teaching hospitals in the Netherlands. All consecutive patients admitted to the three ICUs between 2004 and 2007 were included in the study, except for patients who did not fulfil APACHE II criteria (readmissions, burns, cardiac surgery, younger than 16 years, length of stay less than 8 hours). Data were collected prospectively in the ICU databases. Hospital mortality was the primary endpoint of the study. Off hours was defined as the interval between 10 pm and 8 am during weekdays and between 6 pm and 9 am during weekends. Intensivists, with no responsibilities outside the ICU, were present in the ICU during daytime and available for either consultation or assistance on site during off hours. Residents were available 24 hours a day 7 days a week in two and fellows in one of the ICUs.

**Results:**

A total of 6725 patients were included in the study, 4553 (67.7%) admitted during daytime and 2172 (32.3%) admitted during off hours. Baseline characteristics of patients admitted during daytime were significantly different from those of patients admitted during off hours. Hospital mortality was 767 (16.8%) in patients admitted during daytime and 469 (21.6%) in patients admitted during off hours (*P *< 0.001, unadjusted odds ratio 1.36, 95%CI 1.20–1.55). Standardized mortality ratios were similar for patients admitted during off hours and patients admitted during daytime. In a logistic regression model APACHE II expected mortality, age and admission type were all significant confounders but off-hours admission was not significantly associated with a higher mortality (*P *= 0.121, adjusted odds ratio 1.125, 95%CI 0.969–1.306).

**Conclusions:**

The increased mortality after ICU admission during off hours is explained by a higher illness severity in patients admitted during off hours.

## Introduction

The first few hours after the initial insult are of major importance when treating critically ill patients. Adequate treatment in the first hours has a major impact on outcome. This has been shown for patients with trauma, sepsis, and after major surgery, as well as in various other patient groups [1-4]. Patients can become critically ill at any time, 24 hours a day. Ideally, critical care services have to be organized in such a way that optimal treatment is available to all patients day and night. However, availability and quality of personnel and technology are often different during daytime hours as compared with off hours. As the first hours of treatment are so crucially important, it is conceivable that outcome after intensive care admission depends, at least partially, on the time of the day the patient is admitted. This retrospective multicenter cohort study was performed to evaluate whether outcome is different for patients admitted to the intensive care unit (ICU) during daytime hours as compared with admission during off hours.

## Materials and methods

This retrospective multicenter cohort study was carried out in three intensivist-led ICUs in three different non-academic teaching hospitals in the Netherlands: the 'Onze Lieve Vrouwe Gasthuis' in Amsterdam (OLVG), Gelre Hospitals in Apeldoorn (GH), and 'Reinier de Graaf Groep' in Delft (RDGG). The number of ICU beds in each unit is 16, 10 and 10 respectively. The local ethics committee waived the need for obtaining consent and the need for approval of the study.

### Defining off hours

Daytime hours were defined as the hours between 8 am until 10 pm on weekdays and between 9 am and 6 pm during the weekend. Off hours were defined as the hours between 10 pm and 8 am on weekdays and between 6 pm and 9 am during the weekend.

### Staffing

Intensivists are present in all three units during daytime and make rounds at the bedside at least twice a day. Each day, including weekends, a multidisciplinary meeting is held in which all patients are discussed. During off hours, intensivists on duty are available for consultation 24 hours a day seven days a week and have no other responsibilities apart from ICU-related patient care. During off hours, the intensivist on duty will see all unstable patients and will personally supervise all critical procedures including endotracheal intubation, but intensivists are not routinely present in the ICU during off hours. In two hospitals, RDGG and GH, junior doctors are present in the hospital, available within five minutes, and responsible for ICU patients and airway management. During off hours they may also have responsibilities outside the ICU. In the OLVG, consultants in training to become intensivists, so called fellows, are available exclusively for the ICU around the clock. Nurse to patient ratio is about 1:1.5 during the daytime in all three units. During the nighttime, nurse to patient ratio is 1:2 or 1:2.5 in RDGG and GH. In the OLVG the nightly nurse to patient ratio is on average 1:1.5, never exceeding a ratio of 1:2. Most nurses are registered ICU nurses but all three hospitals also have training programmes for registered nurses training to become ICU nurses. Throughout the study period there were no major changes in the composition of medical or nursing staff.

### Patients

All consecutive patients admitted to the three units between 1 January 2004 and 31 December 2007 were included in the study. Patients were excluded if they were younger than 16 years of age, admitted for less than eight hours, or admitted after cardiac surgery or because of burns (acute physiology and chronic health evaluation score (APACHE) II exclusion criteria) [5]. For patients who were readmitted to the ICU during a single hospital admission episode, only the first ICU admission was taken into account. A patient after elective surgery was defined as a patient admitted to the ICU within a seven-day period after surgery according to the schedule of the operating room. A patient after urgent surgery was defined as a patient admitted to the ICU within seven days after unscheduled surgery. All other patients were defined as medical patients. These definitions are in accordance with the original APACHE II and simplified acute physiology score (SAPS) II definitions and endorsed by the Dutch National Intensive Care Evaluation foundation (NICE) as described below [5,6].

### Data collection

All three units have databases in which the minimal data set as defined by NICE is collected prospectively for each patient [7]. Quality of these data is checked regularly [7]. Demographic data, APACHE II and SAPS II values, expected mortality and hospital discharge status (dead or alive) are among the data collected.

### Study endpoints

Hospital mortality was the endpoint of the study. We calculated crude hospital mortality as well as standardized mortality ratios defined as observed mortality/expected mortality. Logistic regression was also performed analysing different models.

### Statistical analysis

Data were collected prospectively in the ICU databases and analyzed with SPSS 16.0 (Chicago, IL, USA). Normally distributed data were reported as means ± standard deviation (SD). Means were compared using Student's t tests. Nonparametric data were reported as median and interquartile range (IQR). Medians were compared using Mann Whitney U tests. Differences in proportions were compared using Chi square tests, odds ratios and 95% confidence intervals (CI) were also reported. *P *< 0.05 were considered statistically significant.

## Results

A total of 6725 patients were included in the study, 4553 (67.7%) admitted during daytime and 2172 (32.3%) admitted during off hours. Baseline characteristics were not equal for both groups (Table 1). The difference was most marked in patients admitted after elective surgery, that is, off-hour patients after elective surgery were sicker than those admitted during daytime. The difference between the baseline characteristics of daytime and off-hour patients after urgent surgery and medical patients was minimal. We found slight differences in the results between the three hospitals, but these results never conflicted with the overall results (Tables 2 to 4).

**Table 1:** Basic characteristics for all patients

	All patients	Daytime	Off hours	P
Total patient numbers	6725	4553	2172	
Male/Female	3937/2788	2676/1877	1261/911	ns
Mean age, years (SD)	65.1 (16.2)	65.9 (15.3)	63.5 (18.0)	<0.001
Mean APACHE II score at admission (SD)	16.8 (8.7)	16.3 (8.8)	17.9 (9.0)	<0.001
Mean SAPS II score at admission (SD)	37.3 (18.4)	35.7 (18.3)	40.6 (18.2)	<0.001
Mean APACHE II score expected mortality	27.7%	25.8%	31.9%	<0.001
Mean SAPS II score excepted mortality	26.9%	24.8%	31.3%	<0.001
Median ICU LOS (IQR)	2 (1 to 4)	2 (1 to 4)	2 (1 to 5)	ns
Median ICU+Hospital LOS (IQR)	11 (6 to 21)	11 (7 to 21)	11 (5 to 21)	ns
Hospital mortality	1236(18.4%)	767 (16.8%)	469 (21.6%)	<0.001
				
Number of medical patients	3423	2068	1355	
Mean age, years (SD)	62.3 (17.1)	63.2 (16.4)	61.0 (18.0)	<0.001
Mean APACHE II score (SD)	20.0 (9.5)	20.2 (9.4)	19.7 (9.7)	ns
Hospital mortality	880 (25.7%)	548 (26.5%)	332 (24.5%)	ns
				
Number of elective surgery patients	2338	1987	351	
Mean age, years (SD)	68.5 (13.5)	68.2 (13.3)	70.3 (14.9)	0.007
Mean APACHE II score(SD)	12.3 (5.6)	12.0 (15.4)	14.0 (6.2)	0.013
Hospital mortality	186 (8.0%)	133 (6.7%)	53 (15.1%)	<0.001
				
Number of urgent surgery patients	964	498	466	
Mean age, years (SD)	66.6 (17.3)	67.5 (16.3)	65.7 (18.3)	ns
Mean APACHE II score (SD)	16.1 (7.0)	16.6 (7.2)	15.5 (6.8)	ns
Hospital mortality	170 (17.6%)	86 (17.3%)	84 (18.0%)	ns

APACHE = acute physiology and chronic health evaluation score; ICU = intensive care unit; IQR = interquartile range; LOS = length of stay; ns = not significant; SAPS = simplified acute physiology score; SD = standard deviation.

**Table 2:** Basic characteristics for the RDGG

	Day time	Off hours	*P*
Number of patients	2028	576	
Male (%)	1119 (55.2)	328 (56.9)	ns
Mean age, years (SD)	66.6 (15.3)	64.5(18.7)	0.014
Mean APACHE II score (SD)	13.3 (7.4)	14.8 (8.2)	<0.001
Mean SAPS II score (SD)	29.7 (15.9)	35.6(16.6)	<0.001
Mean APACHE II score expected mortality	17.9%	24.2%	<0.001
Mean SAPS II score expected mortality	16.7%	24.0%	<0.001
Median ICU LOS (IQR)	2 (2 to 4)	3 (2 to 5)	<0.001
Median Hospital LOS (IQR)	11 (8 to 19)	10 (5 to 18)	0.001
			
Hospital mortality	263(13.0%)	111(19.3%)	<0.001

APACHE = acute physiology and chronic health evaluation score; ICU = intensive care unit; IQR = interquartile range; LOS = length of stay; ns = not significant; RDGG = Reinier de Graaf Groep, one of the participating hospitals; SAPS = simplified acute physiology score; SD = standard deviation.

**Table 3:** Basic characteristics for the Gelre Hospital

	Day time	Off hours	*P*
Number of patients	1267	754	
Male (%)	781(61.6)	436(57.8)	ns
Mean age, years (SD)	66.5(14.6)	64.7(17.6)	0.014
Mean APACHE II score (SD)	14.7 (6.9)	16.1 (8.0)	<0.001
Mean SAPS II score (SD)	33.3(15.8)	37.9(17.5)	<0.001
Mean APACHE II score expected mortality	19.9%	23.3%	<0.001
Mean SAPS II score expected mortality	22.7%	26.1%	<0.001
Median ICU LOS (IQR)	1 (1 to 4)	2 (1 to 5)	ns
Median hospital LOS (IQR)	12 (7 to 23)	12 (6 to 24)	ns
			
Hospital mortality	180(14.2%)	144(19.1%)	0.005

APACHE = acute physiology and chronic health evaluation score; ICU = intensive care unit; IQR = interquartile range; LOS = length of stay; ns = not significant; SAPS = simplified acute physiology score; SD = standard deviation.

**Table 4:** Basic characteristics for the OLVG

	Day time	Off hours	*P*
Number of patients	1258	842	
Male (%)	776(61.7)	495(61.7)	ns
Mean age, years (SD)	63.9(15.7)	61.8(17.7)	0.005
Mean APACHE II score (SD)	22.5 (8.7)	21.6 (9.1)	0.018
Mean SAPS II score (SD)	47.6(18.5)	46.5(18.1)	ns
Mean APACHE II score expected mortality	42.9%	41.4%	ns
Mean SAPS II score expected mortality	41.9%	40.0%	ns
Median ICU LOS (IQR)	2.3 (1 to 5)	1.8(0.7 to 4.3)	<0.001
			
Median hospital LOS (IQR)	11 (5 to 21)	10 (5 to 20)	ns
			
Hospital mortality	324(25.8%)	214(25.4%)	ns

APACHE = acute physiology and chronic health evaluation score; ICU = intensive care unit; IQR = interquartile range; LOS = length of stay; ns = not significant; OLVG = Onze Lieve Vrouwe Gasthuis, one of the participating hospitals; SAPS = simplified acute physiology score; SD = standard deviation.

Hospital mortality is presented in Tables 1 to 4. We found no mortality difference in urgent surgery and in medical patients between patients admitted during daytime and patients admitted off hours. Standardized mortality ratios are no different for patients admitted during daytime as compared to those admitted during off hours (Tables 5 and 6). Logistic regression analysis confirmed that age, APACHE II expected mortality, and admission type were related to outcome but off-hour admission was not (Tables 7 and 8). Results were similar with SAPS II expected mortality instead of APACHE II expected mortality (not shown).

**Table 5:** Standardized mortality ratios and 95% confidence intervals

	Daytime	Off hours
APACHE II	0.65 (0.60 to 0.71)	0.68 (0.61 to 0.75)
SAPS II	0.68 (0.63 to 0.74)	0.69 (0.62 to 0.76)

APACHE = acute physiology and chronic health evaluation score; SAPS = simplified acute physiology score.

**Table 6:** Standardized mortality ratios and 95% confidence intervals for each separate hospital

	Daytime	Off hours
**RdGG**		
SMR APACHE II	0.73 (0.63 to 0.84)	0.80 (0.64 to 0.99)
SMR SAPS II	0.78 (0.67 to 0.90)	0.80 (0.64 to 1.00)
		
**GH**		
SMR APACHE II	0.66 (0.56 to 0.79)	0.71 (0.58 to 0.85)
SMR SAPS II	0.69 (0.58 to 0.82)	0.70 (0.58 to 0.85)
		
**OLVG**		
SMR APACHE II	0.60 (0.54 to 0.67)	0.61 (0.53 to 0.71)
SMR SAPS II	0.61 (0.55 to 0.69)	0.64 (0.55 to 0.73)

APACHE = acute physiology and chronic health evaluation score; GH = Gelre Hospital, one of the participating hospitals; OLVG = Onze Lieve Vrouwe Gasthuis, one of the participating hospitals; RDGG = Reinier de Graaf Groep, one of the participating hospitals; SAPS = simplified acute physiology score; SMR = standardized mortality ratios.

**Table 7:** Logistic regression model

Parameters in the model	Wald	P	Adjusted odds ratio (95% CI)
APACHE II expected mortality	755.9	<0.001	67.4 (50.0 to 91.1)
Age (per year)	118.0	<0.001	1.030 (1.024 to 1.035)
Admission type	14.8	0.001	
Admission type (1)	0.001	0.969	0.995 (0.772 to 1.283)
Admission type (2)	9.15	0.002	1.377 (1.119 to 1.695)
Off hours admission	1.89	0.121	1.125 (0.969 to 1.306)

Admission type (1): urgent surgery vs elective surgery = reference.

Admission type (2): medical vs elective surgery = reference.

APACHE = acute physiology and chronic health evaluation score; CI = confidence interval.

**Table 8:** Logistic regression models for each separate hospital

Parameters in the model	P	Adjusted OR (95% CI)
**RdGG**		
APACHE II expected mortality	<0.001	202 (108 to 372)
Age (per year)	<0.001	1.04 (1.03 to 1.05)
Admission type	<0.001	
Admission type (1)	0.003	1.67 (1.19 to 2.34)
Admission type (2)	0.257	0.77 (0.50 to 1.21)
Off hours admission	0.266	1.19 (0.87 to 1.60)
		
**GH**		
APACHE II expected mortality	<0.001	70 (37 to 130)
Age (per year)	<0.001	1.03 (1.02 to 1.04)
Admission type	<0.001	
Admission type (1)	0.167	1.29 (0.90 to 1.84)
Admission type (2)	0.494	1.17 (0.75 to 1.83)
Off hours admission	0.508	1.10 (0.83 to 1.45)
		
**OLVG**		
APACHE II expected mortality	<0.001	51 (32 to 83)
Age (per year)	<0.001	1.02 (1.01 to 1.03)
Admission type	0.187	
Admission type (1)	0.590	1.12 (0.75 to 1.68)
Admission type (2)	0.391	0.81 (0.51 to 1.31)
Off hours admission	0.462	1.09 (0.87 to 1.37)

Admission type (1): urgent surgery vs elective surgery = reference.

Admission type (2): medical vs elective surgery = reference.

APACHE = acute physiology and chronic health evaluation score; CI = confidence interval; GH = Gelre Hospital, one of the participating hospitals; OLVG = Onze Lieve Vrouwe Gasthuis, one of the participating hospitals; OR = odds ratio; RDGG = Reinier de Graaf Groep, one of the participating hospitals.

## Discussion

In this retrospective cohort study with prospectively collected data we found no difference in case-mix adjusted hospital mortality between ICU patients admitted during daytime as compared with those admitted during off hours. Differences in hospital mortality could all be explained by differences in disease severity.

Several authors have studied the difference in outcome between patients admitted during daytime hours as compared with off hours in ICU patients, all in retrospective cohort studies, all defining off hours differently [8-16]. No formal meta-analysis has been performed, but the literature has been reviewed recently [8,12]. In three studies, an increased mortality was found for patients admitted during off hours even after adjustment for potential confounders [9,11,15]. In the remaining six studies no increased mortality was seen for patients admitted during off hours [8,10,12-14,16]. One author even found better outcome for patients admitted during off hours [12]. From looking at these studies one cannot judge the quality of off-hour ICU care in general, but we can conclude that off-hour care is not necessarily inadequate. For ICU managers it is important to know how to maintain adequate quality of care round the clock. In the present study, the definition of off hour was based on the presence or absence of the intensivist in the ICU. Future studies should investigate whether the organisational model with intensivists present during the day and residents supervised by intensivist during off hours is adequate enough to avoid a 'quality gap' during off hours.

This study has several important limitations. Although data collection was prospective, the study hypothesis was formulated later, which makes this essentially a retrospective study. However, the division between the two groups is solely based on time and not the subject of subjective assessment or bias. Different definitions of the off-hour interval showed similar results. If one assumes, as was done in the present study, that the first few hours of intensive care are more decisive than the time that follows, a difference in the quality of care between daytime and off hours may also be reflected in final outcome. If, however, the first few hours of critical illness are not so important, and for example off hour care is always suboptimal, most patients will experience this presumed suboptimal care during all off-hour shifts in their intensive care episode. In this way the burden of suboptimal care will be divided equally among daytime admitted patients and off hour-admitted patients and no difference in outcome will be found. Considering the very low standardized mortality ratios in the present study, in our opinion, this is unlikely. However we cannot prove that the quality of care does not differ between daytime and off hours. Excluding patients that were in the ICU for less than eight hours may have excluded the most severely ill patients that died soon after admission. APACHE II expected mortality calculation is validated only after an eight-hour observation period [5]. This is not so for SAPS II but it is conceivable that mortality prediction even by SAPS II will be less accurate after a short observation time [6]. The present study relies heavily on assessment of illness severity by APACHE II and SAPS II scoring. We felt that we needed at least two separate assessments of illness severity for all patients in the study and thus decided to exclude patients for whom only a probably imprecise SAPS II score was applicable. The higher mortality in the elective surgery group may be attributed to the classification of the admission type. Patients admitted within seven days of surgery were classified according to their original type of surgery (elective/emergency). However, the reason for ICU admission (especially outside office hours) most probably does not correspond to the surgical indication. A patient admitted during daytime immediately after scheduled surgery is different from a patient admitted during off hours within seven days after previous scheduled surgery. So actually, we probably are looking at two different groups within the surgical group. The study was performed in three ICUs with intensivist available 24 hours a day seven days a week. Concerns about off-hour quality of care are mostly about ICUs without 24-hour intensivist coverage. The present study is not informative on this subject. Finally, hospital mortality is only a small part of quality of care. We did not study patient, family or nurse satisfaction, resource utilization, faults, (near) accidents, or adverse events as related to off hours, all of which could be significantly different during off hours.

## Conclusions

In this retrospective multicenter cohort study we found, after correction for illness severity, no difference in hospital mortality between patients admitted to the ICU during daytime hours as compared with patients admitted during off hours.

## Key messages

• In this study in three intensivist-led ICUs, daytime was defined by the time period in which intensivists are routinely present in the unit and off hours was defined by the time period in which intensivists are not routinely present in the ICU but available for consultation by telephone and present if necessary for critical procedures or unstable patients.

• Unadjusted hospital mortality was higher after off-hour ICU admission as compared with admission during daytime.

• However, after adjustment for illness severity, hospital mortality for patients admitted during off hours and patients admitted during daytime were equal.

• We speculate that if intensivists are continuously present in the ICU during daytime and present when necessary during off hours this is sufficient to avoid a quality gap during off hours.

## Abbreviations

APACHE: acute physiology and chronic health evaluation score; CI: confidence interval; GH: Gelre Hospital, one of the participating hospitals; ICU: intensive care unit; IQR: interquartile range; NICE: National Intensive Care Evaluation; OLVG: Onze Lieve Vrouwe Gasthuis, one of the participating hospitals; RDGG: Reinier de Graaf Groep, one of the participating hospitals; SAPS: simplified acute physiology score; SD: standard deviation.

## Competing interests

The authors declare that they have no competing interests.

## Authors' contributions

The idea for the study was conceived by all authors together at a reunion party. IAM analyzed the data from the RDGG and the aggregate data, wrote the first version of the manuscript and together with PES and MvS-V supervised the preparation of the final manuscript. JIvdS and RJB analyzed data of the OLVG and contributed in the preparation of the final manuscript. JHR and PES analyzed data of the GH and contributed in the preparation of the final manuscript.
